# Monthly Summary

**Published:** 1875-12

**Authors:** 


### MONTHLY SUMMARY.

*On the Diseases Ascribed to Dentition, and their Pathological Admissibility.*—Dr. L. M. Pollitzer is inclined to differ very decidedly from the views held by A. Vogel regarding dentition and the diseases connected with it. He maintains that in the great majority of cases there is no such thing as catarrhal stomatitis accompanying the eruption of each tooth, the process going on so gradually and easily as not to be a source of inflammatory irritation, while the ulcers on the tongue, constituting stomatiti sulcerosa are due to friction on the tooth, which has penetrated the gum. Nor does he think worthy of greater consideration the idea that the increase of the fluids of the mouth during dentition averts the danger of brain symptoms. He is much more inclined to attribute diarrhœa at this period to the ingestion of unsuitable food than to the irritation of dentition. He cannot recognize the periodicity and association with the eruption of the teeth, which Vogel maintains regarding various skin diseases. The various diseases occurring during dentition may all be observed at other times, and we should not attribute them to this cause unless all other known causes are absent. As to the great reflex irritability said to exist in childhood, and to be increased during dentition, he argues that it is astonishing to observe how well a young child will bear severe operations, very painful pathological conditions, and other powerful influences, without the development of reflex irritability; and why should we attribute to the comparatively easy and unimportant process of dentition an increased irritability which is not claimed for important physical, histological, and chemical changes going on in various important organs?—*Med. Record.*

---

*Third Dentition.*—In the Hospital for Incurables, Paris, an instance of third dentition took place in a nun aged ninety years. She had, on previous occasions, once when forty-seven and again when sixty-three years old, cut one new tooth.—*Med. and Surg. Journal.*

*Syphilis of Parents as Affecting the Offspring.*—Dr. Drysdale, in notes to *The Medical Press and Circular*, ranks himself on the side of those who hold that syphilitic children can only be born when the mother is syphilitic. This view, opposed by the authority of Trousseau, Diday, Benton, Depaul, Lee, Acton, and other writers of great note, is sustained by Notta, Berkeley, Gascoyen, Cullerier, and Owse, of Christiania, and others Drysdale cites the following instances in support of the latter opinion: A gentleman contracted syphilis in 1850. He Married some years afterwards, and now has three blooming children. Another gentleman had syphilis in 1851, and his wife contracted syphilis from him directly. She had no children. Four years later the gentleman married again, and now has a fine family of sons and daughters. Two gentlemen of his acquaintance contracted syphilis to his knowledge, and have had large families of healthy children The case is not so where the woman has contracted syphilis. A lady contracted syphilis from her future husband, who bit her lip. He was suffering from a secondary affection of the mouth. She married, but had no children by this husband, who died in three years. A few years later the lady again married, and gave birth to a dead child. In view of such facts as he has collected, he feels warranted in assuring his male patients that if they have been free from syphilis *some two years* they will not have syphilitic children should they become fathers.—*Medical Record.*

---

*Curious Brain Lesions.*—The St. Louis *Clinical Record* contains a remarkable case of brain lesion. A man named Waters, living near Leavenworth, Kansas, died from morphia a short time ago, and as it was known that he had had a habit of running wires, and even nails through his own skull during his former residence in the penitentiary, a careful autopsy was performed. Two openings were found in the skull, one, which the prisoner was known to have made with a brad-awl, near the inferior posterior angle of the right parietal, and another near the superior posterior angle of the same bone. A wire was found in the brain. It had been introduced through the upper opening, and just missing the superior longitudinal sinus, had pierced to the base of the brain, a little in front of the fissure of Sylvius. It is stated that the corpus striatum was not wounded. A nail, one and three-fourth inches in length, was found lying beside the wire. Although wires had been removed during life from the lower aperture, no trace of their course was discovered. Though the patient had shown a suicidal tendency he did not appear insane, except for this habit, and could do his work with correctness and understanding.

*Neuralgia Treated by Nerve-stretching.*—Mr. Callender, of London, reports the case of a man who had undergone amputation of the forearm twice—the second time owing to imperfect healing and a painful condition of the stump. The second wound healed, but he was liable to attacks of pain in the stump. About seven weeks before his presenting himself to Mr. C., he struck the end of the stump, and since then the pain had greatly increased. The limb was cold, the skin glazed and of a dusky color, the surface tissue was inelastic, and the skin was moved with stiffness over the subjacent fascia. He complained of twitchings in the forearm as well as in the arm, though in the latter to a less degree ; but his chief distress arose from the constant pain, sometimes burning or shooting, but liable to become suddenly much more severe, and at times to be almost unbearable. Various measures for his relief were resorted to, but without success, and an operation was decided upon. The median nerve was cut down upon, and found to be thickened in itself and in its surroundings. After dissecting it away from the tissues about it for the distance of about an inch, it was forcibly stretched by pulling it downwards for about three-quarters of an inch. The wound was then closed. Some pain occurred on the following day, but none afterwards. One month after the operation the wound had healed, the arm was warm and had regained its natural appearance, and the patient was in all respects well.—*The London Lancet.*

---

*On the Relief of Toothache by Bicarbonate of Soda.*—Dr. Dyce Duckworth records in *The Practitioner* a case of severe toothache in a boy, which he attempted to relieve by rubbing the cheek with chloroform : by putting some on cotton wool inside the auditory meatus, by plugging the tooth with cotton wool saturated with chloroform, also with carbolic acid, but to no purpose. He next tried a solution of bicarbonate of soda, which was quickly followed by complete relief.

The Pathology of saliva is as yet, so far as I know, observes Dr. Duckworth, an uncultivated field, but I believe, if it be looked for, that this secretion will be more often found to possess an acid reaction, than is generally believed or thought, and I also think that useful therapeutical hints may be gathered by the employment, at various intervals, of litmus paper in the mouth, in many cases of dyspepsia and chronic disease.—*Monthly Review of Dental Surgery.*

---

*Dental Neuralgia.*—Dr. J. Sawyer says, in the *Practitioner*, "I have rarely found gelseminum fail to give decided and lasting relief in cases of neuralgic pains in the face and jaws, associated with carious teeth. I have usually given fifteen mimims of the tincture every six hours."

